# Risk factor identification for delayed excretion in pediatric high-dose methotrexate therapy: a machine learning analysis of real-world data

**DOI:** 10.3389/fphar.2025.1662718

**Published:** 2025-09-17

**Authors:** Chengyu Zhou, Yali Qian, Yao Xue, Liucheng Rong, Yu Wan, Kaiqiang Leng, Hongjun Miao, Feng Chen, Yongjun Fang, Xuhua Ge

**Affiliations:** ^1^ Pediatric Intensive Care Unit, Children’s Hospital of Nanjing Medical University, Nanjing, China; ^2^ Department of Pediatrics, The Second People’s Hospital of Changzhou Affiliated to Nanjing Medical University, Nanjing, China; ^3^ Department of Hematology, Children’s Hospital of Nanjing Medical University, Nanjing, China; ^4^ Department of Pharmacy, Children’s Hospital of Nanjing Medical University, Nanjing, China; ^5^ Jiangsu Key Laboratory of Children’s Major Disease Research, Children’s Hospital of Nanjing Medical University, Nanjing, China

**Keywords:** methotrexate, delayed excretion, pediatric, machine learning, predictive model

## Abstract

**Purpose:**

This study was to identify risk factors associated with delayed methotrexate (MTX) excretion in pediatric patients receiving high-dose MTX (HDMTX) therapy based on real-world data, and to develop and evaluate a predictive model.

**Methods:**

Clinical data were retrospectively collected from 1,485 pediatric HDMTX chemotherapy cycles at the Children’s Hospital affiliated with Nanjing Medical University between 2021 and 2023. Key predictive variables were identified by Least Absolute Shrinkage and Selection Operator (LASSO) regression, Random Forest (RF), and Support Vector Machine Recursive Feature Elimination (SVM-RFE), and then incorporated into predictive models for MTX delayed excretion using Logistic Regression (LR), Naive Bayes (NB), Support Vector Machine (SVM), and Extreme Gradient Boosting (XGBoost). Bootstrap was employed to internally validate these models and identify the best-performing one, and then SHapley Additive exPlanations (SHAP) values were utilized to provide both global and local interpretations.

**Results:**

Among the 1,485 pediatric HDMTX chemotherapy cycles, 26.1% were associated with delayed MTX excretion. Serum creatinine (Scr), total drug dose (Dose), alkaline phosphatase (ALP), creatine kinase (CK), blood urea nitrogen (Urea), gamma-glutamyl transferase (GGT), hemoglobin (HB), and height were identified as key predictors of delayed excretion. Internal validation showed that the XGBoost model performed best, with an accuracy of 0.780, an F1 score of 0.669, an area under the Receiver Operating Characteristic curve (AUROC) of 0.842, and a Brier score of 0.136. Decision Curve Analysis (DCA) also demonstrated favorable clinical utility. SHAP analysis revealed that Scr was the most important risk factor for delayed MTX excretion in the XGBoost model. This XGBoost model has been translated into a convenient tool to facilitate its utility in clinical settings.

**Conclusion:**

The XGBoost model demonstrated good predictive performance and clinical utility for delayed MTX excretion in pediatric patients.

## Introduction

Methotrexate (MTX) administered intravenously at doses of ≥500 mg/m^2^ or >20 mg/kg is defined as high-dose methotrexate (HDMTX) (2019). HDMTX significantly increases the serum concentration of MTX, allowing it to penetrate the blood-brain barrier and reach poorly vascularized solid tumors, which makes it widely used in first-line chemotherapy for diseases such as Acute Lymphocytic Leukemia (ALL) and Non-Hodgkin Lymphoma (NHL) (Prodduturi and Bierman, 2012). HDMTX therapy can improve the overall prognosis of patients with hematological malignancies; however, the MTX doses used are potentially lethal. While these doses inhibit tumor growth, they can also damage proliferating normal tissues, leading to toxic events such as acute liver and kidney injury, bone marrow suppression, mucositis, gastrointestinal issues, fever, and infections (2019). Studies have shown that delayed MTX excretion further increases the risk of these toxicity events (Yu et al., 2024).

Currently, most related studies suggest that age, body surface area, and drug dosage are the primary influencing factors for MTX excretion delay (Chen et al., 2020; Zhang et al., 2022; Yu et al., 2024). Additionally, creatinine clearance, drug interactions, and genetic polymorphisms also impact the *in vivo* clearance process of MTX (Faganel Kotnik et al., 2011; Kawase et al., 2016; Tran and Herrington, 2016; Umerez et al., 2017; Hamada et al., 2018; Hui et al., 2019; Ishizaki et al., 2020; Ueda et al., 2020; Yang et al., 2020; Ferracini et al., 2021; Ebid et al., 2022; Mao et al., 2022). However, there are several discrepancies among different studies, and many of these studies rely on traditional statistical methods to screen risk factors and build predictive models. These methods often suffer from poor sensitivity and specificity, resulting in fluctuating prediction outcomes and significant reliance on the researcher’s data analysis expertise.

This study was based on real-world data and employed machine learning methods to construct a more stable and predictive risk model. The primary aims were to explore the predictive factors for delayed MTX excretion in pediatric patients, identify children at risk of MTX excretion delay as early as possible, and to provide a basis for precision treatment, thereby reducing the incidence of toxicity events.

## Materials and methods

### Study subjects

This was a retrospective cohort study. The subjects were pediatric patients aged 0–16 years with hematological malignancies who received HDMTX treatment at the Children’s Hospital of Nanjing Medical University between 1 January 2021, and 31 December 2023. A total of 1,485 chemotherapy cycles were included in this study. The study was approved by the Ethics Committee of the Children’s Hospital of Nanjing Medical University (No. 202411021-1), with a waiver of informed consent.

Due to the dynamic nature of growth and development in pediatric patients, weight, height, and body surface area may vary considerably between chemotherapy cycles. In addition, laboratory assessments (e.g., complete blood count, liver and kidney function) are standardly repeated prior to each cycle to rule out contraindications. In practice, baseline values for both demographic and laboratory parameters are not static between chemotherapy cycles within the same individual. To reflect the clinical reality and capture cycle-specific risk, we modeled each treatment cycle as an independent unit, even for patients contributing multiple cycles.

The study subjects met the following inclusion criteria: ⅰ) All chemotherapy cycles were part of a sequential four-cycle HDMTX + 6-MP treatment regimen. Each cycle was spaced approximately 2 weeks apart, provided that the patient was in complete remission after prior chemotherapy, had completed baseline assessments, and had no contraindications to chemotherapy (otherwise, treatment was delayed). No other chemotherapy regimens were administered between consecutive HDMTX+6-MP cycles. ⅱ) Patients concurrently received intrathecal administration of a triple regimen consisting of MTX, dexamethasone (Dex), and cytarabine (Ara-C). ⅲ) Each chemotherapy cycle had available MTX plasma concentration data at 24 h, 48 h, and 72 h post-infusion. ⅳ) The underlying disease was either ALL or NHL, and the MTX dose administered was approximately 3–5 g/m^2^. Patients were excluded if they experienced severe drug hypersensitivity reactions, or were unable to complete the HDMTX chemotherapy cycle for any reason, or had incomplete data.

Delayed MTX excretion was defined as plasma MTX concentration >1 μmol/L at 48 h post-infusion (C_48h_), or ≥0.2 μmol/L at 72 h post-infusion (C_72h_) Based on this definition, patients were categorized into the delayed excretion group and the non-delayed excretion group. All patients were treated according to the standardized protocol outlined in the Chinese Society of Clinical Oncology (CSCO) (2019). At our institution, the discharge criterion was defined as an MTX plasma concentration <0.2 μmol/L.

### Data collection

Clinical data were collected through the hospital’s electronic medical record system. The collected data included: a) Demographic information: age, height, weight, body surface area (BSA), and gender; b) Diagnosis and treatment information: chemotherapy cycle number, MTX dose per unit of body surface area (g/m^2^), total MTX dosage, and disease diagnosis; c) MTX plasma concentrations: measured at baseline (before MTX infusion), 24 h, 48 h, and 72 h post-infusion for each chemotherapy cycle, and additional measurements at extra time points performed on-demand; d) Laboratory tests: complete blood count, liver and kidney function, and biochemical indicators.

MTX plasma concentrations were determined using the Enzyme-Multiplied Immunoassay Technique (EMIT) assay kit, manufactured by Siemens Healthcare Diagnostics Inc., United States. The lower limit of detection for MTX was 0.1 μmol/L.

### Research methodology

#### Feature selection

Three machine learning methods—Least Absolute Shrinkage and Selection Operator (LASSO) regression, Random Forest (RF), and Support Vector Machine - Recursive Feature Elimination (SVM-RFE) — were applied to the original dataset to perform feature selection. These methods are capable of identifying the most relevant variables while reducing model complexity and overfitting risk. The detailed descriptions and parameter settings for LASSO, RF, and SVM-RFE are provided in Supplementary Table S1. A consensus-based approach was used to determine the final set of key predictive features.

#### Model training

The key predictive features identified were used as input variables to develop machine learning models. Four different machine learning algorithms—Logistic Regression (LR), Naive Bayes (NB), Support Vector Machine (SVM), and Extreme Gradient Boosting (XGBoost) — were employed to construct predictive models for delayed excretion of HDMTX in pediatric patients (Descriptions and parameter settings for the LR, NB, SVM, and XGBoost algorithms are provided in Supplementary Table S2). To ensure comparability across models, all models were trained using the same set of input features. Subsequently, grid search and randomized search approaches were applied to identify the optimal hyperparameters for each model based on the training data. The area under the receiver operating characteristic curve (AUROC) was used as the primary evaluation metric during hyperparameter tuning.

#### Internal model validation

Bootstrap resampling with 1,000 iterations was used to perform internal validation of the developed models. The performance of the four machine learning models was evaluated in terms of discrimination, calibration, and clinical utility using a comprehensive set of metrics, including: accuracy, sensitivity, specificity, positive predictive value (PPV), negative predictive value (NPV), F1 Score, AUROC, Brier Score, and Decision Curve Analysis (DCA). These metrics facilitated the selection of the optimal predictive model.

#### Model interpretation

The best-performing model was interpreted using SHapley Additive exPlanations (SHAP) through the following steps: a) Feature Contribution Analysis: A SHAP value was assigned to each feature to reflect its contribution to the model’s prediction. b) SHAP Summary Plot: A summary plot was generated to visualize the relative importance of each feature and to show how the magnitude and direction of feature values influence the predicted risk of delayed MTX excretion. c) SHAP Dependency Plots: For each important feature, a dependency plot was created to explore the relationship between the feature value and the predicted outcome.

#### Web-based prediction tool

To facilitate the clinical application of this predictive model, we have developed a web-based application. When the corresponding feature values are provided, the application can return the probability of MTX excretion, with a waterfall plot and a force plot.

### Statistical analysis

Data processing was performed using R software (version 4.4.2). Continuous variables that followed a normal distribution were expressed as mean ± standard deviation (x ± s), and intergroup comparisons were conducted using the independent samples t-test. For continuous variables with non-normal distribution, data were presented as median (first quartile, third quartile) [M (Q1, Q3)], and group comparisons were performed using the Mann-Whitney U test. Categorical variables were expressed as counts and percentages (n, %). Comparisons between groups were carried out using the chi-square (χ^2^) test or Fisher’s exact test. A two-sided P value <0.05 was considered statistically significant.

## Results

### Patient baseline characteristics

A total of 1,485 HDMTX chemotherapy cycles from 408 pediatric patients were included in this study. Among these, 387, 389, 358, and 351 cycles corresponded to the first, second, third, and fourth treatment cycles, respectively. 388 cycles were identified as delayed excretion cases, resulting in a delayed excretion rate of 26.1%. Descriptive statistics of the covariates between the two groups are presented in Table 1.

**TABLE 1:** The baseline characteristics of patients in the non-delayed excretion group and the delayed excretion group.

Characteristics	The non-delayed excretion group (n = 1,097)	The delayed excretion group (n = 388)	*P* value
Demographic information			
Age (year)	5.0 (3.2,8.1)	7.4 (4.1,11.0)	<0.0001
Height (m)	1.12 (0.96,1.30)	1.27 (1.05,1.47)	<0.0001
Weight (kg)	19.0 (14.5,26.0)	24.3 (17.0,35.0)	<0.0001
BSA (m^2^)	0.77 (0.61,1.01)	0.95 (0.70,1.15)	<0.0001
Sex			0.044
Male	614 (55.9%)	240 (61.9%)	
Female	483 (44.1%)	148 (38.1%)	
Treatment information			
Dose (mg)	2,770 (2000,4,000)	3,505 (2400,5295)	<0.0001
Dose/BSA (mg/m^2^)	3,421 (2992,4971)	4,227 (3015,4992)	0.005
Period			0.002
Period 1	265 (24.2%)	122 (31.4%)	
Period 2	279 (25.4%)	110 (28.4%)	
Period 3	272 (24.8%)	86 (22.2%)	
Period 4	281 (25.6%)	70 (18%)	
Laboratory tests			
WBC (×10^9^/L)	3.68 (2.85,5.04)	3.77 (2.89,5.52)	0.125
N (×10^9^/L)	1.49 (1.02,2.32)	1.71 (1.14,2.69)	0.002
HB (g/L)	104 (94,113)	100 (91,109)	<0.0001
PLT (×10^9^/L)	228 (168,307)	230 (158,319)	0.925
Urea (mmol/L)	3.48 (2.61,4.46)	3.20 (2.23,4.20)	0.002
UA (μmol/L)	229 (192,271)	240 (202,282)	0.002
Scr (μmol/L)	24.0 (19.9,28.9)	27.0 (22.0,34.1)	<0.0001
Cys-c (mg/L)	1.01 (0.870,1.14)	0.98 (0.85,1.14)	0.225
K (mmol/L)	4.23 (4.00,4.51)	4.17 (3.88,4.48)	0.005
Na (mmol/L)	140 (139,141)	141 (139,142)	0.002
Cl (mmol/L)	105.4 (104.0.107.0)	105.2 (103.7,106.7)	0.044
Ca (mmol/L)	2.52 (2.41,2.61)	2.50 (2.39,2.61)	0.076
Mg (mmol/L)	0.89 (0.84,0.94)	0.88 (0.83,0.93)	0.058
P (mmol/L)	1.72 (1.57,1.84)	1.69 (1.51,1.83)	0.042
eGFR (mL/min/1.73 m^2^)	222.0 (197.4,253.0)	219.7 (193.4,260.9)	0.666
ALT (U/L)	54 (27,100)	60 (29,119)	0.025
AST (U/L)	27 (21,37)	29 (22,46)	0.049
ALP(U/L)	177 (143,219)	160 (129,197)	<0.0001
GGT (U/L)	41 (22,77)	49 (25,95)	<0.0001
LDH (U/L)	257 (223,297)	260 (225,304)	0.213
CK (U/L)	33 (24,46)	30 (21,44)	0.001
CK-MB (U/L)	17 (13,22)	15 (12,20)	0.001
HBDH (U/L)	178 (155,208)	179 (153,212)	0.665
TP (g/L)	62.1 (58.9,65.7)	62.6 (58.83,66.4)	0.235
ALB (g/L)	41.9 (3.1,44.5)	41.3 (38.3,43.9)	0.017
GLO (g/L)	20.3 (18.0,23.0)	21.4 (18.5,24.4)	<0.0001
A/G	2.03 (1.75,2.38)	1.91 (1.66,2,24)	<0.0001
GLU (mmol/L)	4.55 (4.15,4.94)	4.58 (4.19,5.04)	0.097
TBIL (μmol/L)	7.19 (5.51,9.64)	7.27 (5.57,10.07)	0.301
DBIL (μmol/L)	2.85 (2.16,3.81)	2.99 (2.22,4.34)	0.015
PA (g/L)	0.19 (0.15,0.23)	0.20 (0.16,0.24)	0.183

BSA, body surface area; Dose, total dose of methotrexate administered; Dose/BSA, dose of methotrexate per unit of body surface area; Period, the sequence number of chemotherapy cycles; WBC, white blood cell count; N, absolute neutrophil count; HB, hemoglobin concentration; PLT, platelet count; Urea, blood urea nitrogen; UA, uric acid level; Scr, serum creatinine; Cys-c, cystatin C; K, potassium concentration; Na, sodium concentration; Cl, chloride concentration; Ca, calcium concentration; Mg, magnesium concentration; P, phosphorus concentration; eGFR, estimated glomerular filtration rate; ALT, alanine aminotransferase; AST, aspartate aminotransferase; ALP, alkaline phosphatase; GGT, gamma-glutamyl transferase; LDH, lactate dehydrogenase; CK, creatine kinase; CK-MB, creatine kinase MB, fraction; HBDH, alpha-hydroxybutyrate dehydrogenase; TP, total protein; ALB, albumin; GLO, globulin; A/G, albumin to globulin ratio; GLU, glucose level; TBIL, total bilirubin; DBIL, direct bilirubin; PA, prealbumin.

^a^ BSA, was estimated using a weight-based formula: Weight≤ 30 kg, BSA (m2) = Weight × 0.035 + 0.1; Weight> 30 kg, BSA (m2) = (Weight-30) × 0.02 + 1.05.

^b^ eGFR, was estimated using a Schwartz formula: eGFR [ml/(min·1.73 m2) ] = K × Height (cm) × 88.4/Scr (μmol/L). The constant K in the Schwartz formula varies by age and sex as follows: 0–28 days, K = 0.33; 28 days to 1 year, K = 0.45; 2–12 years, K = 0.55; above 12 years: K = 0.77 for males, and = 0.55 for females.

### Feature selection using machine learning methods

**FIGURE 1:**
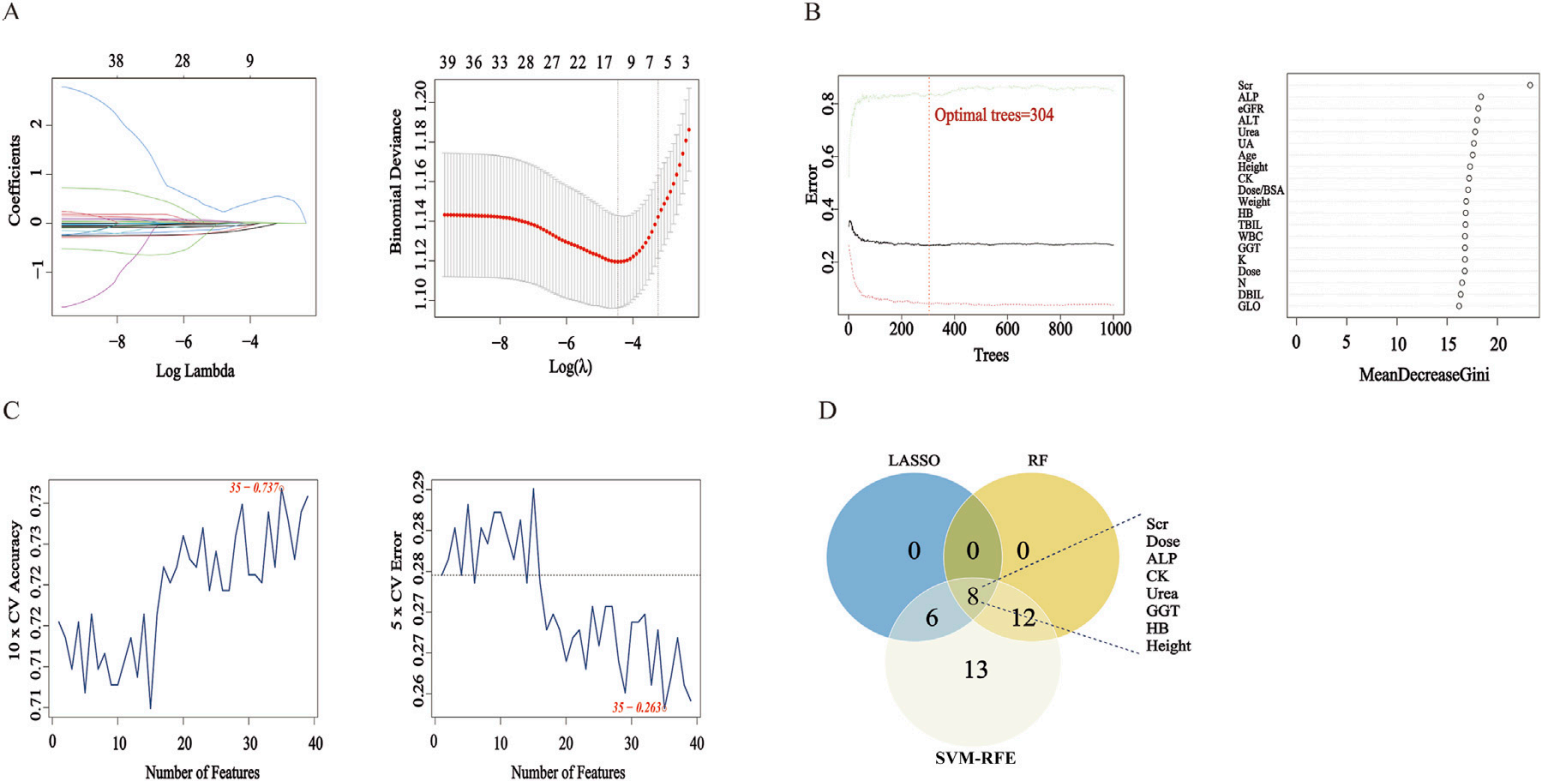
Feature Selection Results Using LASSO, RF, and SVM-RFE. Note: **(A)** LASSO Regression Coefficient Path Plot and LASSO Cross-Validation Curve. **(B)** RF Number of Trees vs Error Plot and RF Feature Importance Ranking Plot. **(C)** SVM-RFE Accuracy vs Number of Features Plot and SVM-RFE Generalization Error vs Number of Features Plot. **(D)** Venn Diagram: The Venn diagram displays the intersection of feature sets selected by LASSO, RF, and SVM-RFE.

Each algorithm identified a distinct set of variables, as shown in Figures 1A–C. This consensus-based feature selection approach (Figure 1D) yielded a final set of 8 key features: Serum Creatinine (Scr), Dose, Alkaline Phosphatase (ALP), Creatine Kinase (CK), Blood Urea Nitrogen (Urea), Gamma-glutamyl Transferase (GGT), Hemoglobin (HB), and Height. These eight core features were subsequently used to construct predictive models for delayed MTX excretion using four different machine learning algorithms.

### Prediction model construction and internal validation

Using the eight key features selected through feature selection, four machine learning models were developed based on LR, NB, SVM, and XGBoost. To assess model performance and ensure internal validity, bootstrap resampling with 1,000 iterations was performed.

**FIGURE 2:**
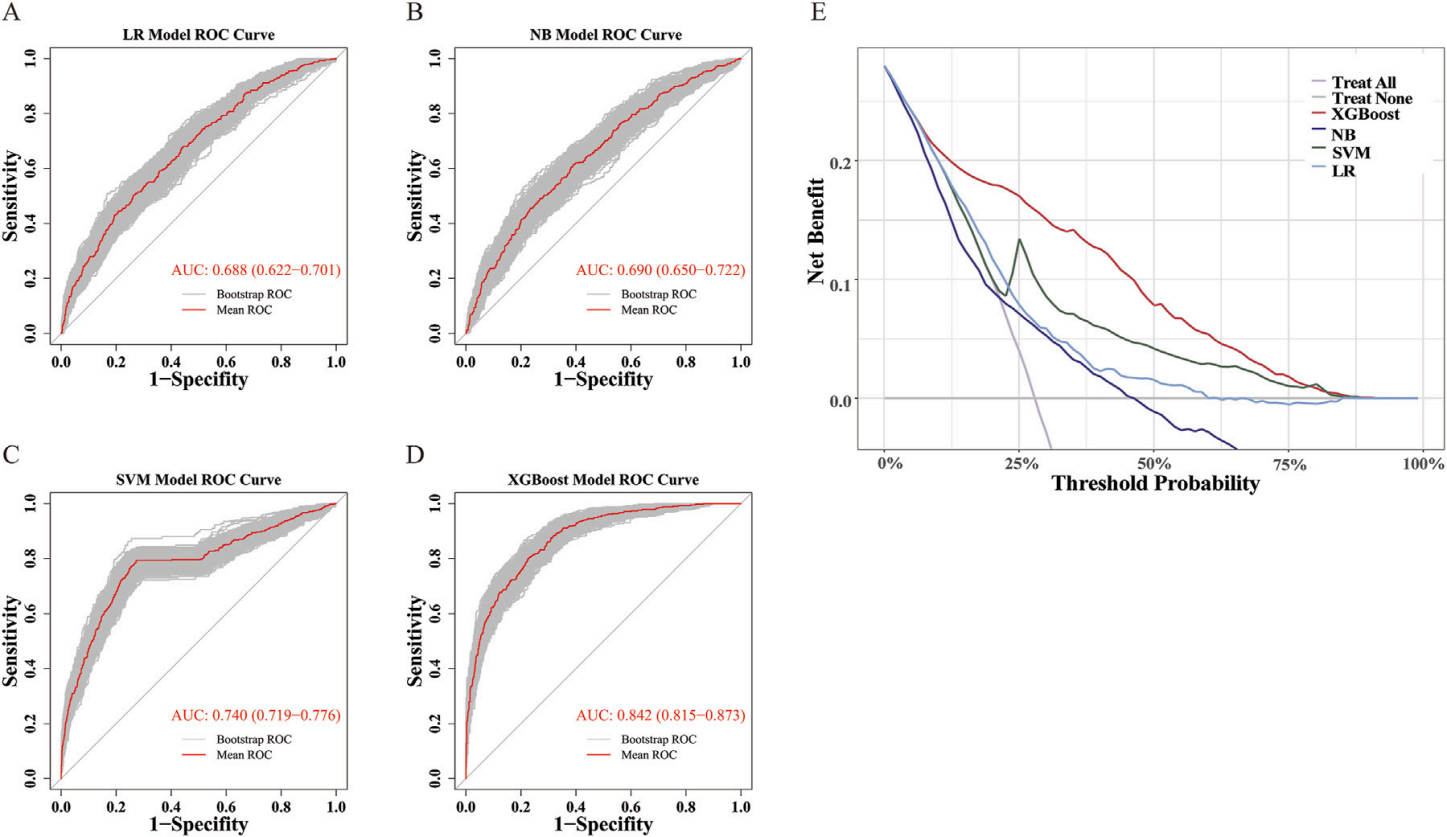
ROC and DCA Curves. **(A–D)** ROC curves for LR, NB, SVM, and XGBoost Models. ROC curve was performed via 1,000 bootstrap iterations. The 45-degree diagonal line represents the baseline for random classification. The red line represents the mean ROC of 1,000 bootstrap iterations. The grey lines represents the actual ROC of 1,000 bootstrap iterations. **(E)** DCA Curves for LR, NB, SVM, and XGBoost Models.

Among the four models, the XGBoost model demonstrated the best overall performance across all validation metrics (Table 2; Figures 2A–D), with an AUC of 0.842 (95%CI: 0.815–0.873). The calibration curve demonstrated that the probability of MTX excretion delay predicted by the XGBoost model was relatively well matched with the actual measurement (Supplementary Figure S1).

**TABLE 2:** Performance metrics of the predictive models in internal validation.

	Accuracy (95%CI)	Specificity (95%CI)	Sensitivity (95%CI)	NPV (95%CI)	PPV (95%CI)	F1 score (95%CI)
LR	0.672 (0.648,0.695)	0.792 (0.766,0.816)	0.437 (0.383,0.471)	0.738 (0.710,0.763)	0.502 (0.455,0.550)	0.461 (0.420,0.499)
NB	0.609 (0.585,0.634)	0.802 (0.773,0.828)	0.378 (0.343,0.416)	0.609 (0.579,0.638)	0.613 (0.565, 0.659)	0.468 (0.430,0.502)
SVM	0.745 (0.722,0.766)	0.898 (0.876,0.916)	0.529 (0.489,0.568)	0.728 (0.701,0.754)	0.786 (0.744,0.823)	0.632 (0.597,0.665)
XGBoost	0.780 (0.758,0.800)	0.907 (0.886,0.924)	0.577 (0.536,0.617)	0.774 (0.748,0.798)	0.796 (0.754,0.832)	0.669 (0.633,0.701)

Additionally, sensitivity analysis was performed using only the first chemotherapy cycle from each patient. A new predictive model was developed based on this subset. As shown in Supplementary Figure S2, XGBoost again emerged as the optimal model, and there was no significant difference in performance between this model and the one trained on all treatment cycles (DeLong test, P = 0.967).

In addition, DCA showed that the XGBoost model had the widest applicable threshold range (approximately 10%–80%). Within most of this range (approximately 10%–75%), the XGBoost model provided the highest benefit among all four models, reaching up to 0.2 (Figure 2E). This indicates that the XGBoost model offers the greatest clinical utility in terms of decision-making compared to the other models.

### Model interpretation using SHAP

**FIGURE 3:**
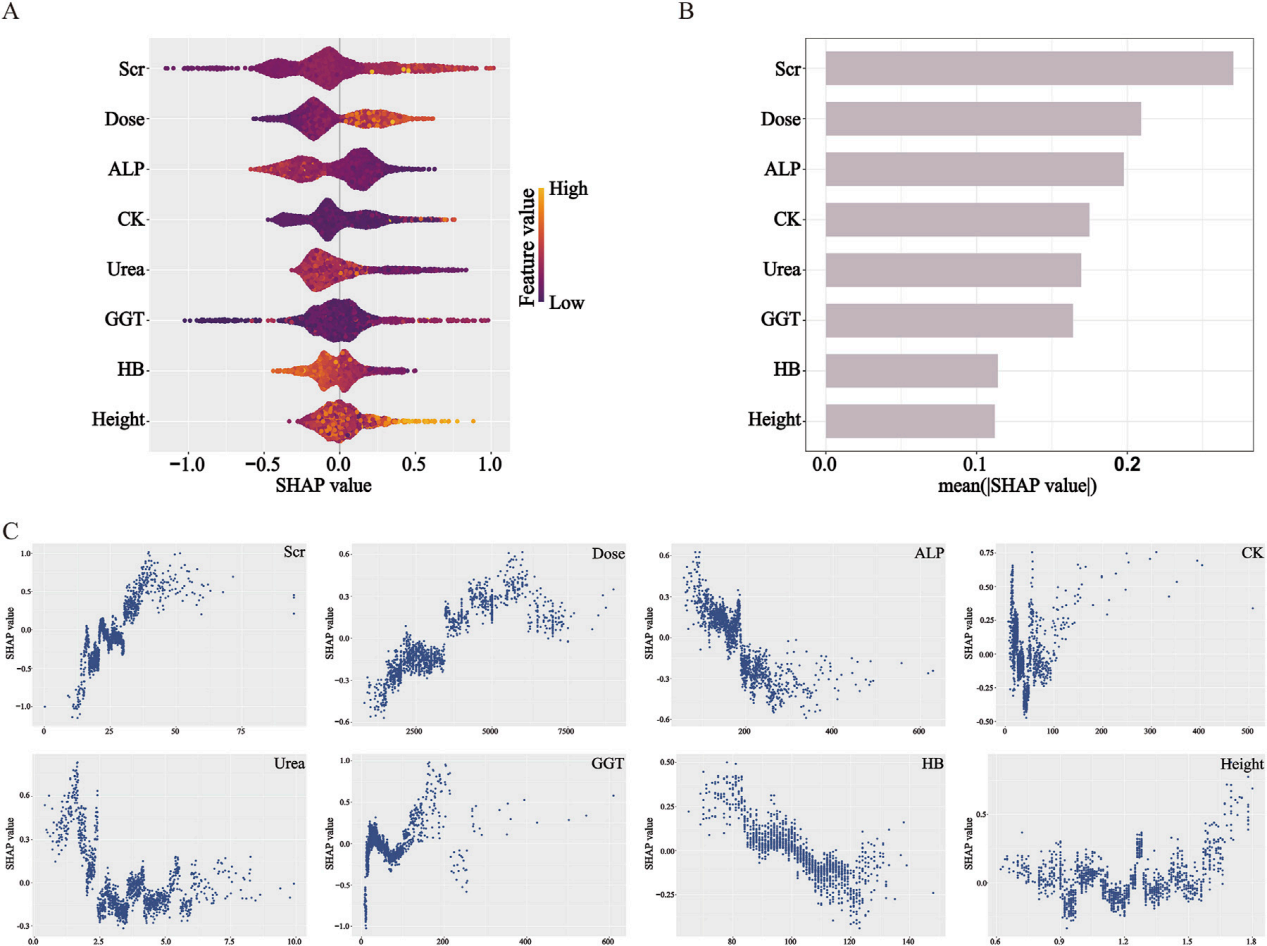
SHAP analysis elucidates feature influence and direction in the XGBoost model. **(A)** Beeswarm plot: features are ranked by impact magnitude; dot color indicates feature value (yellow: high, purple: low). **(B)** Bar plot: features are ordered by mean |SHAP|, representing overall importance. **(C)** Dependence plots: illustrate how feature values affect SHAP values, indicating contribution direction. SHAP values >0 increase predicted risk.

The contribution of the eight selected predictive features in the XGBoost model was interpreted using SHAP analysis, as illustrated in the Summary Plot (Figures 3A,B) and Dependence Plots (Figure 3C). Among the eight predictors, Scr was identified as the most influential feature in predicting delayed MTX excretion. Dose and ALP followed in terms of importance. Higher levels of Scr, higher MTX dose, and lower levels of ALP were associated with an increased risk of delayed excretion. In comparison, the other five variables—CK, Urea, GGT, HB, and height—showed relatively weaker influences on the model’s predictions.

### Clinical application of the prediction model

**FIGURE 4:**
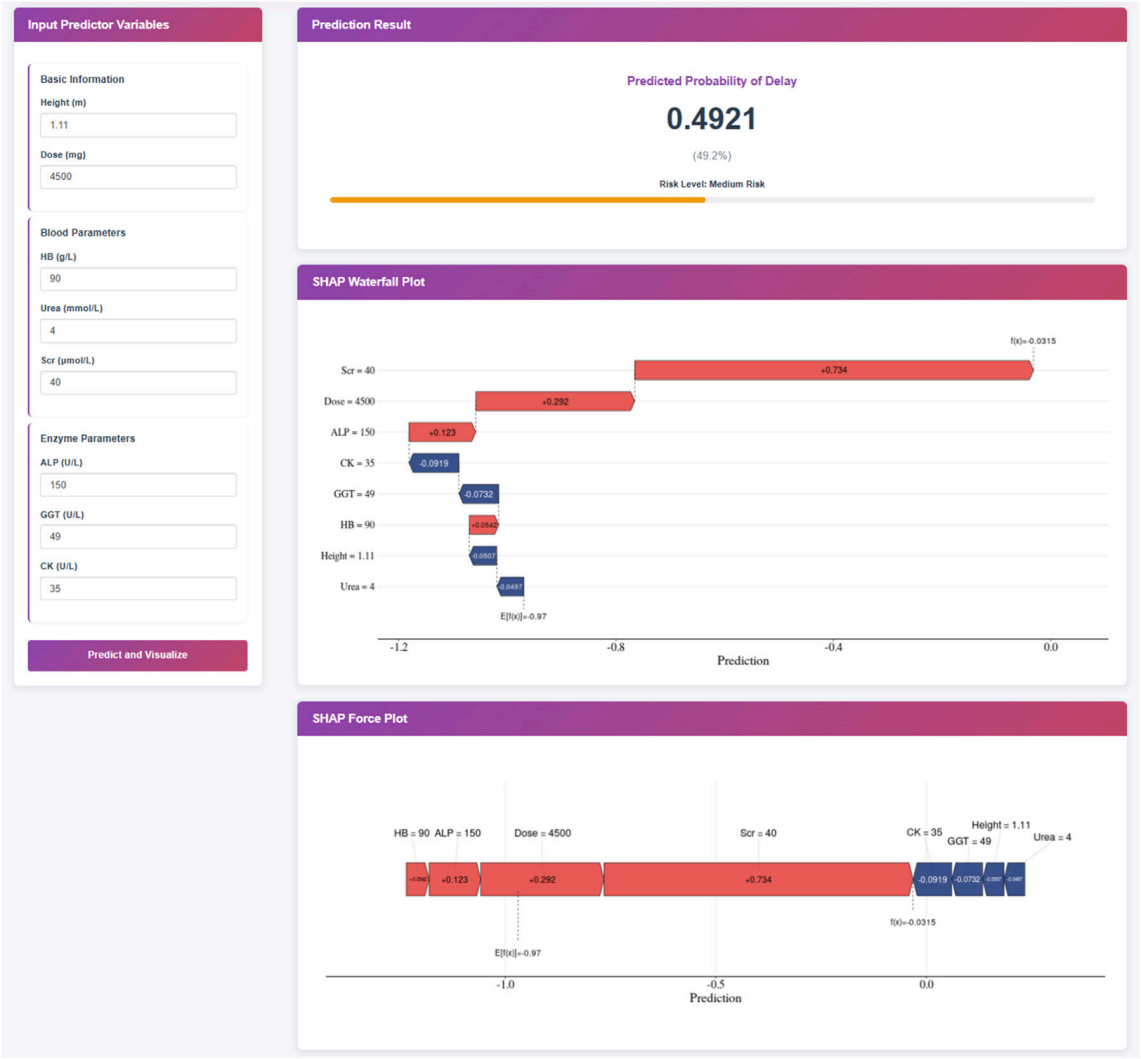
Clinical Application of the XGBoost Prediction Model. Note: This XGBoost prediction model, built on eight clinically relevant features, offers a user-friendly interface for predicting the risk of delayed MTX excretion in pediatric patients. Upon entering the actual values of the eight features, the application automatically displays a predicted probability of 50.5% for MTX excretion delay. Furthermore, individual patient explanations are visualized through waterfall plots and force plots, which clearly illustrate the key features influencing the prediction: blue-colored features drive the prediction toward “no delay” while red-colored features push the prediction toward “delay”.

To facilitate the practical use of the final XGBoost prediction model in clinical settings, we developed a web-based application (Figure 4). When the actual values of the eight predictive features are entered into the tool, the application automatically calculates and displays the individual risk of delayed MTX excretion for each pediatric patient. In addition to the predicted probability, the web tool generates two interpretable visualizations: Waterfall plot and Force plot. The web application is publicly accessible at https://delay-prediction-njch.ndemo.net.

## Discussion

HDMTX therapy is associated with significant antitumor efficacy; however, it also carries substantial toxicity risks (Mosleh et al., 2023). Given that children have less mature organ development and lower tolerance to drug toxicity compared to adults, they are at a higher risk of severe adverse events, including mortality. Delayed MTX excretion is typically defined based on whether plasma MTX concentrations exceed predefined threshold levels at specific time points after infusion. However, elevated MTX levels are usually detected only after delayed excretion has already occurred, limiting opportunities for early intervention. This study was a retrospective analysis of pediatric patients with ALL or NHL who received HDMTX therapy. Leveraging real-world data and machine learning techniques, we identified key predictors of delayed MTX excretion from demographic, dosing, treatment cycle, and laboratory variables. A risk prediction model was subsequently developed to identify high-risk patients before HDMTX administration. By enabling early risk stratification, this model supports the delivery of individualized, precision-based interventions to potentially reduce the incidence of delayed excretion and associated toxicities.

Most existing studies on delayed MTX excretion have traditionally relied on conventional statistical methods for feature selection and predictive model development. These traditional approaches, such as linear regression, require the fulfillment of numerous assumptions—linearity, independence, normality, homoscedasticity, among others—before they can be applied effectively. When dealing with smaller datasets, simple linear models are often the most reasonable choice due to their interpretability and straightforward implementation. However, these models’ expressiveness becomes notably limited as dataset sizes increase. In contrast, machine learning models can overcome many of the limitations imposed by traditional statistical models and incorporate techniques like regularization to mitigate overfitting (Christodoulou et al., 2019; Chen et al., 2023). Consequently, in this study, we opted to utilize machine learning methods for model construction. Machine learning models, especially more complex ones, typically exhibit superior predictive performance but are often criticized as “black boxes” due to their opaque decision-making processes. To address this interpretability issue, we employed SHAP, which leverages concepts from cooperative game theory to explain individual predictions made by machine learning models. By calculating each feature’s contribution to the prediction outcome, SHAP values provide a means to assign optimal credit allocation and local explanations. This approach allows us to better understand how the XGBoost model arrives at its decisions, thereby enhancing transparency and trustworthiness in clinical applications.

In this study, the feature selection analyses using LASSO, RF, and SVM-RFE consistently identified eight key predictors of delayed MTX excretion: Scr, total MTX dose, ALP, CK, Urea, GGT, HB, and height. These eight variables were then incorporated into four machine learning models—LR, NB, SVM, and XGBoost—for predictive modeling. Based on internal validation, the XGBoost model demonstrated superior performance compared to the other models. Therefore, we focused on interpreting the XGBoost model using SHAP to explore the contribution of each predictor in individual risk assessment.

The SHAP analysis conducted on the XGBoost model revealed that Scr had the highest contribution to the prediction of delayed MTX excretion. The dependence plot illustrated higher Scr values were consistently associated with increased model-predicted risk of delayed MTX excretion, which was consistent with the statistical associations observed in previous studies (Xu et al., 2014; Mei et al., 2018; Zhan et al., 2022). From a pathophysiological perspective, MTX and its metabolites are primarily eliminated through glomerular filtration and proximal tubular secretion into the primary urine, followed by excretion via urine (Mosleh et al., 2023). When renal function, particularly glomerular filtration rate (GFR), is compromised, the clearance of MTX decreases. Scr is a common indicator of glomerular filtration. Notably, SHAP reflects model-learned associations, not causation—while higher Scr correlates with higher predicted risk, this does not prove it directly causes delayed excretion.

The SHAP analysis revealed that total MTX dose had the second-highest contribution to predicting delayed MTX excretion, following Scr. Higher doses of MTX were associated with an increased model-predicted risk of delayed excretion. Additionally, ALP was identified as another significant predictor in the model. Lower ALP levels were associated with a model-predicted higher risk of delayed MTX excretion. This observation may reflect underlying anemia or malnutrition due to the primary disease. As malnutrition is known to affect the function of various tissues and organs, it could potentially influence drug metabolism efficiency. However, the model itself cannot establish a causal link.

CK, Urea, and GGT showed relatively moderate contributions in the SHAP feature importance ranking. The SHAP dependence plot for CK revealed a non-linear relationship between CK levels and the predicted risk of delayed MTX excretion: when CK < 50 U/L, lower values were associated with model-predicted risk scores of delayed excretion; in the intermediate range (50–100 U/L), changes in CK levels had minimal impact on the predicted risk; when CK > 100 U/L, higher values were linked to the predicted elevated risk of delayed MTX excretion. Low CK levels may be attributed to several factors, including the younger age of pediatric patients—whose baseline CK activity is naturally lower—or underlying disease-related malnutrition. However, this interpretation represents a clinical hypothesis independent of the model’s findings. The dependence plot for Urea demonstrated that: when Urea levels were below the normal reference range (1.79–6.43 mmol/L), lower values correlated with higher risk of delayed MTX excretion; within the normal range, variations in Urea had no significant effect on the model-predicted risk scores. The dependence plot of GGT indicated: within the normal reference range (8–61 U/L), changes in GGT levels did not significantly alter the predicted risk; when GGT exceeded the normal range, it may increase the risk of delayed MTX excretion. Elevated GGT levels are often indicative of hepatic or biliary dysfunction, such as impaired bile flow or liver damage (Lu and Association, 2022). Since MTX undergoes extensive hepatic metabolism, this may partially explain the association patterns identified by the model.

HB and height demonstrated relatively weaker contributions to the predictive performance of the XGBoost model. However, their SHAP dependence plots still revealed meaningful associations with the risk of delayed MTX excretion. The SHAP dependence plot for HB showed that lower HB levels were associated with a higher model-predicted risk of delayed MTX excretion. The dependence plot for height indicated a non-linear relationship: when height was below 150 cm, variations in height had minimal impact on the predicted risk; when height exceeded 150 cm, increased height was associated with higher predicted risk scores in the model. Generally, taller patients tend to have larger BSA and higher MTX clearance rates (Mei et al., 2018; Hui et al., 2019; Chen et al., 2020; Yang et al., 2020). However, they also receive higher total doses of MTX based on BSA-adjusted dosing protocols. The nonlinear patterns identified by the model may reflect a dynamic interplay between these two opposing effects: in children shorter than 150 cm, the increased clearance associated with greater height may offset the effects of higher drug doses, resulting in no significant net change in the risk of delayed excretion; in contrast, once height exceeds 150 cm, the increase in drug load may outpace the compensatory rise in clearance, leading to a greater likelihood of delayed excretion. This observation requires further investigation and validation in independent cohorts.

This was a large-sample, single-center retrospective cohort study to date investigating risk factors for delayed MTX excretion. A major strength of our work lies in the development of a web-based prediction tool based on the final XGBoost model, which allows clinicians to conveniently input patient-specific features and obtain real-time individualized risk predictions along with interpretable visualizations (e.g., waterfall and force plots). This user-friendly interface enhances the practical applicability of our findings in daily clinical practice. Despite these strengths, several limitations should be acknowledged. First, we did not include drug-drug interactions in our analysis, as the usage rates of potentially interacting medications were all below 10%. This low prevalence may reflect clinical efforts to actively avoid prescribing drugs that could potentially delay MTX excretion. Second, due to the retrospective nature of the study, genetic testing was not routinely performed at the time of treatment, and data on urine output and urine pH were frequently missing. As a result, polymorphisms in drug-metabolizing genes, urine volume, and urinary pH were not included in the analysis. Additionally, all patients in this cohort received standardized hydration, urinary alkalinization, and leucovorin rescue according to the Expert Consensus on High-Dose Methotrexate with Calcium Folinate Rescue for the Treatment of Malignant Tumors. As a result, variables such as hydration rate/volume, degree of alkalinization, and timing/dose of leucovorin were not included in the model. However, the omission of these factors may introduce unmeasured confounding bias. For instance, inadequate fluid intake and low urine output are well-established risk factors for MTX-induced nephrotoxicity, which directly delay drug excretion (Howard et al., 2016; Chen et al., 2020). If not accounted for, the model may underestimate risk in dehydrated patients while overestimating the importance of markers such as serum creatinine (Scr)—which itself can be influenced by volume status. Furthermore, urinary pH is critical for MTX solubility; when pH falls below 7.0, the risk of crystalline nephropathy increases substantially (Bezabeh et al., 2012; Kawaguchi et al., 2021). The current model did not capture this key physiological mechanism, potentially limiting its accuracy in predicting delays caused by intratubular precipitation. Concomitant use of interacting medications—such as proton pump inhibitors (PPIs), which inhibit renal tubular secretion of MTX, or other nephrotoxic agents—can significantly alter MTX pharmacokinetics (Howard et al., 2016). Quantifying the precise impact of these unmeasured confounders is challenging, but their absence may lead to an over-optimistic assessment of model performance. This could restrict the model’s generalizability across diverse clinical settings. The model may be most applicable to patients receiving optimal hydration, without significant drug interactions or additional renal insults. Future studies should aim to address these gaps by conducting multi-center prospective investigations, expanding the range of sample types, and incorporating a broader range of covariates to further refine and validate the predictive performance of the model and improve the model’s utility in supporting clinical decision-making.

In conclusion, machine learning models can serve as reliable tools for predicting the occurrence of delayed MTX excretion during HDMTX therapy in pediatric patients. Among the models evaluated, the XGBoost model, constructed using key predictors including Scr, total drug dose, ALP, CK, Urea, GGT, HB, and height, demonstrated the best predictive performance. This study provides a practical approach for early identification of high-risk patients for MTX excretion delay, thereby supporting personalized treatment decisions. By enabling timely interventions, the model has the potential to reduce the incidence of delayed excretion and associated toxicities, ultimately improving both the safety and adherence of chemotherapy in pediatric oncology.

## Data Availability

The raw data supporting the conclusions of this article will be made available by the authors, without undue reservation.

## Supplementary material

SUPPLEMENTARY FIGURE S1 Calibration curve for the models. **(A)** Calibration curve of the LR model; **(B)** Calibration curve of the NB model; **(C)** Calibration curve of the SVM model; **(D)** Calibration curve of the XGBoost model. The slope of Ideal (diagonal) was 1, which represents the ideal curve; Apparent represents the uncalibrated prediction curve and Bias-corrected represents the calibration prediction curve.

SUPPLEMENTARY FIGURE S2 ROC Curves of the machine learning models in the cycle 1. **(A-D)** ROC curves for LR, NB, SVM, and XGBoost Models. ROC curve was performed via 1,000 bootstrap iterations. The 45-degree diagonal line represents the baseline for random classification. The red line represents the mean ROC of 1,000 bootstrap iterations. The grey lines represents the actual ROC of 1,000 bootstrap iterations.
