# Supplementary material for: Risk factor identification for delayed excretion in pediatric high-dose methotrexate therapy: a machine learning analysis of real-world data
Source: Front Pharmacol. 2025 Sep 17;16:1662718. doi: 10.3389/fphar.2025.1662718 (PMC12483887; doi:10.3389/fphar.2025.1662718)
Supplement: Supplementary file 1 [file Table1.docx]

Supplementary Table S1 Machine Learning Methods for Feature Selection

| **Methods** | **Introduction** |
| --- | --- |
| Least Absolute Shrinkage and Selection Operator (LASSO) Regression | LASSO is a feature selection method that enhances conventional multiple linear regression by introducing a penalty term. This penalty shrinks the coefficients of less important variables toward zero, effectively removing them from the model. As a result, LASSO produces a more parsimonious and interpretable model with stronger explanatory power. One of the key strengths of LASSO lies in its stability and efficiency in variable selection, especially in high-dimensional datasets. By reducing the number of irrelevant or redundant features, LASSO helps mitigate issues such as multicollinearity and overfitting. Unlike traditional stepwise selection methods such as forward selection or backward elimination, LASSO regression offers a more efficient approach. It is particularly advantageous when dealing with datasets involving a large number of variables relative to the sample size, enabling effective feature selection even with relatively small cohorts. |
| Random Forest (RF) | RF is an ensemble learning model based on the bootstrap aggregating (bagging) method, and it can be applied to both classification and regression tasks. The fundamental concept of RF involves the following key steps: 1) Construction of Multiple Decision Trees: A large number of decision trees are built independently. Each tree is trained on a random bootstrap sample drawn from the original dataset, and at each node split, only a random subset of features is considered. This randomness helps reduce model variance. 2) Aggregation of Predictions: For regression tasks, the final prediction is obtained by averaging the predictions of all individual trees. For classification tasks, the majority voting mechanism is used to determine the final class label. RF demonstrates strong generalization performance, especially when dealing with complex, high-dimensional datasets involving a large number of features, it is also robust to overfitting. |
| Support Vector Machine-Recursive Feature Elimination (SVM-RFE) | SVM is a boundary-based classification method that aims to find an optimal hyperplane in the feature space, which maximally separates different classes. By maximizing the margin between the hyperplane and the nearest data points from each class, SVM achieves strong generalization performance on the training data. SVM-RFE is a sequential backward selection algorithm based on the maximum-margin principle of SVM. It works by iteratively training an SVM model on the full feature set, and then removing the least informative feature. The model is then retrained on the remaining features, and this process is repeated recursively until an optimal subset of features is identified—those that yield the best model performance. This approach effectively reduces feature redundancy and enhances model interpretability and predictive accuracy. |
