# Supplementary figures and images for: Risk factor identification for delayed excretion in pediatric high-dose methotrexate therapy: a machine learning analysis of real-world data
Source: Front Pharmacol. 2025 Sep 17;16:1662718. doi: 10.3389/fphar.2025.1662718 (PMC12483887; doi:10.3389/fphar.2025.1662718)

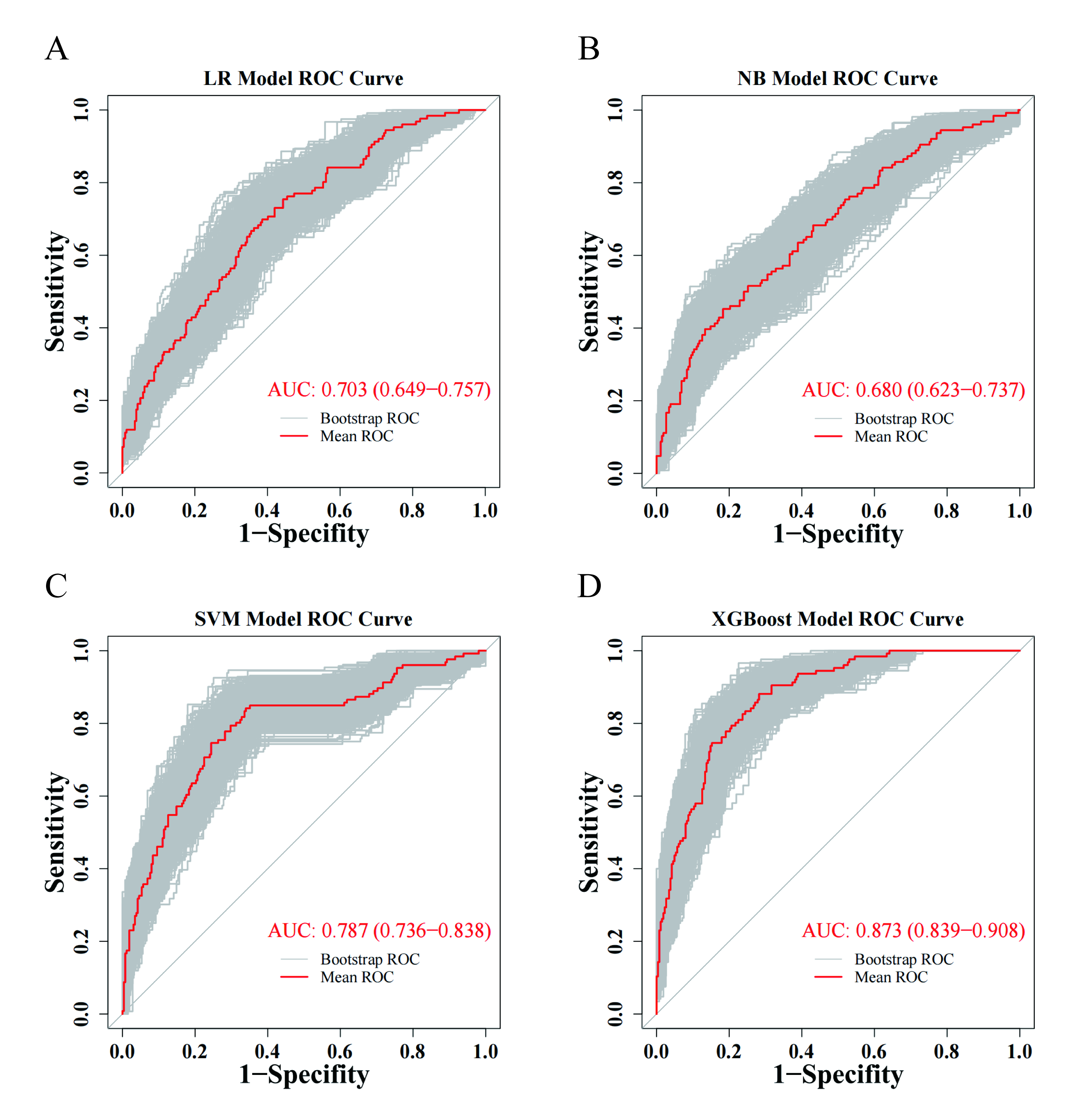

Supplement: Supplementary file 2 [file Image2.tif]

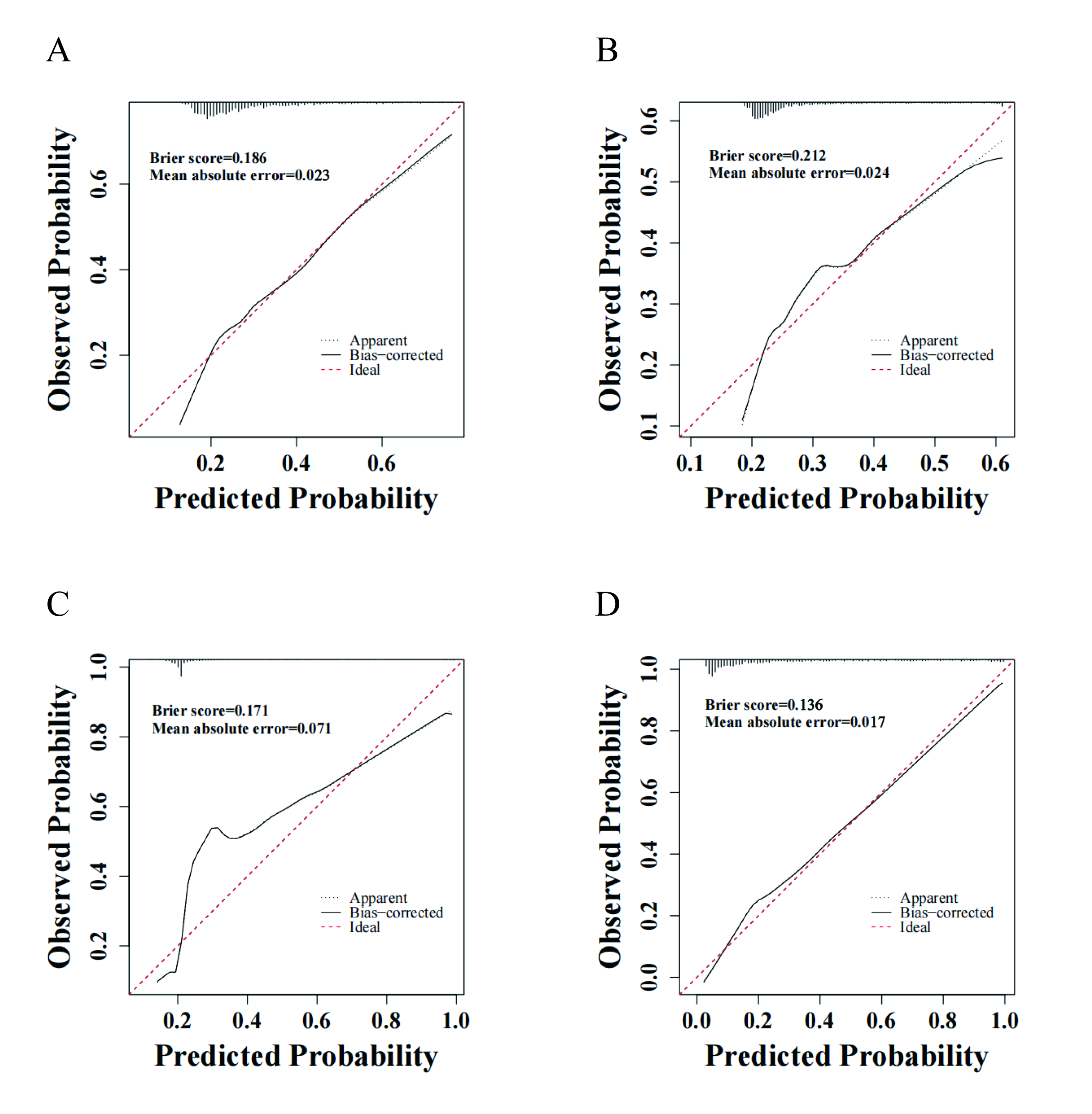

Supplement: Supplementary file 3 [file Image1.tif]
