# Supplementary material for: Risk factor identification for delayed excretion in pediatric high-dose methotrexate therapy: a machine learning analysis of real-world data
Source: Front Pharmacol. 2025 Sep 17;16:1662718. doi: 10.3389/fphar.2025.1662718 (PMC12483887; doi:10.3389/fphar.2025.1662718)
Supplement: Supplementary file 4 [file Table2.docx]

Supplementary Table S2 Machine Learning Methods for Model Training

| **Methods** | **Introduction** |
| --- | --- |
| Logistic Regression (LR) | LR is one of the most commonly used machine learning algorithms, particularly for predicting the probability of an event occurring. Due to its simplicity and interpretability, LR is often employed as a baseline model for comparison with more complex predictive models. Computational Workflow of LR: 1) LR uses the sigmoid function to transform a linear combination of input features into a value between 0 and 1, representing the probability of the positive class. A threshold—typically set at 0.5—is applied to classify predicted probabilities. Values above the threshold are assigned to the positive class, while those below are assigned to the negative class. 2) The performance of the model is evaluated using the log loss (cross-entropy loss), which quantifies the discrepancy between predicted probabilities and actual binary labels. 3) To prevent overfitting and reduce multicollinearity, the original loss function is extended by adding a regularization term. The model parameters are then optimized using gradient descent, aiming to minimize the regularized loss function and obtain the final set of coefficients that best fit the data. |
| Naive Bayes (NB) | NB is a classification algorithm based on Bayes’ theorem and the assumption of feature conditional independence. Given a training dataset, NB assumes that each feature contributes independently to the probability of a particular class outcome. By learning, NB can achieve the probability distributions of features under each class. Computational Workflow: 1) The prior probability of each class is calculated based on its frequency in the training dataset. In this study, the two classes correspond to the occurrence or non-occurrence of delayed MTX excretion. 2) For each feature, the probability distribution under each class is estimated. 3) For a given set of feature values, the posterior probability of each class is computed. The class with the highest posterior probability is selected as the predicted outcome. |
| Support Vector Machine (SVM) | SVM is a widely used supervised learning algorithm that can be applied to both classification and regression tasks. Its core idea is to find an optimal hyperplane in the feature space that best separates samples from different classes, with the goal of maximizing the margin—the distance between the hyperplane and the nearest data points from each class. Computational Workflow of SVM: 1) An appropriate kernel function is selected based on the nature of the data. The regularization parameter C is optimized through techniques such as cross-validation. 2) Using the selected kernel and tuned value of C, the SVM model is trained on the dataset. 3) The performance of the SVM model is evaluated using validation metrics. Based on these results, further parameter tuning or kernel adjustments may be performed to enhance predictive performance. |
| Extreme Gradient Boosting (XGBoost) | XGBoost is a highly efficient and scalable implementation of the Gradient Boosting Decision Tree (GBDT) algorithm, which belongs to the family of ensemble learning methods. As a boosting algorithm, its emphasis on computational speed, memory efficiency, and numerical optimization, making it one of the most widely used algorithms for tasks such as classification, regression, and ranking. Computational Workflow of XGBoost: 1) A simple initial model is constructed. The residuals—i.e., the differences between the actual values and the model’s predictions—are computed. 2) A new decision tree is trained to predict the residuals from the previous model. This tree is then added to the ensemble in a way that reduces the overall prediction error. 3) Steps 2 is repeated multiple times, with each newly added tree focusing on correcting the errors made by the existing ensemble. This iterative process continues until a predefined number of trees are built or the prediction error converges to an acceptable level. The final model is a weighted sum of all individual trees, capable of approximating complex data distributions with high accuracy. |
